# Perceived Paternal Involvement, Relationship Satisfaction, Mothers' Mental Health and Parenting Stress: A Multi-Sample Path Analysis

**DOI:** 10.3389/fpsyt.2020.578682

**Published:** 2020-11-02

**Authors:** Francine deMontigny, Christine Gervais, Tamarha Pierce, Geneviève Lavigne

**Affiliations:** ^1^Laboratory at the Heart of Families, Department of Nursing Science, Center of Research and Studies in Family Intervention, Université du Québec en Outaouais, Gatineau, QC, Canada; ^2^Department of Psychology, Université Laval, Québec City, QC, Canada; ^3^Center of Research and Studies in Family Intervention, Université du Québec en Outaouais, Gatineau, QC, Canada

**Keywords:** maternal depression, parenting stress, paternal involvement, dyadic adjustment, parental alliance, maternal mental health

## Abstract

Research has demonstrated the short- and long-term impacts of maternal mental health and well-being on children's emotional and behavioral outcomes. It is thus important to better understand the antecedents of maternal depression and stress. The aim of this study was to determine whether the contribution of perceived paternal involvement to account for mothers' depression and parental stress was mediated by relationship factors such as parenting alliance and dyadic adjustment. A second aim was to determine whether these relationships hold equally true in mothers of infants and young toddlers (0–24 months) and mothers of older children (25 months and older). Cross-sectional data were collected from 447 mothers. Mothers reported on their perceptions of paternal involvement with childcare responsibilities, dyadic adjustment, parenting alliance, parenting stress, and depression. Multi-sample path modeling analyses were conducted. Results revealed that perceived paternal involvement was positively related to both dyadic adjustment and parental alliance, that parenting alliance was negatively related to all three subscales of parenting stress and mothers' depression but that dyadic adjustment was negatively related to parenting distress (one subscale of parenting stress) and mothers' depression. Results from the multi-sample analyses indicated that the pattern of relationships was the same in the two groups, but that the model was not invariant. The most notable difference was that parenting alliance did not significantly account for depression in the mothers of younger children. Correlates of maternal mental health and well-being identified in this study could be useful when designing psychological interventions for mothers and fathers.

## Introduction

A growing body of research clearly indicates the contribution of maternal mental health and well-being to children's behavioral and emotional outcomes (1–6), but fewer studies have focused on the relational predictors of maternal mental health and well-being. Partner social support (7–11), and more importantly, fathers' active participation in childcare responsibilities (12, 13), appear to be important predictors of maternal mental health and well-being. Furthermore, recent research (14–16) suggests that elements of mothers' relationships with their partner might contribute to explaining the link between paternal involvement and maternal mental health and well-being. In the present study, we thus posited the hypothesis that mothers' satisfaction with their relationship with their partner, operationalized as dyadic adjustment and parenting alliance, mediates the relationship between mothers' perceptions of paternal involvement and two dimensions of maternal mental health and well-being: parenting stress and depression.

A number of studies have highlighted the relationship between partner support and women's anxiety and depression in the antenatal period (7, 9, 10) as well as in the postpartum period (9). Yet the father's role involves more than supporting the mother. Recently, the constructs of paternal involvement (involvement with parenting responsibilities) have also been studied in relation to mothers' mental health and well-being. For instance, significant negative associations have been reported between paternal involvement and maternal parenting stress (12). However, the extent to which mothers' perceptions of paternal involvement help account for their mental health and well-being remain a little-known field of study.

The changes that happen in a couple's life when they become parents have been shown to influence a number of relationship factors, such as marital satisfaction (17, 18), which in turn contribute to explaining maternal mental health and well-being (19–21). For instance, Clout and Brown (22) showed that women's dyadic satisfaction late in pregnancy was a significant predictor of their depression and anxiety levels 4–6 months postpartum, with high dyadic satisfaction being associated with better outcomes. Thus, to better understand what may explain maternal mental health and well-being, it is important to consider the quality of the marital relationship.

Parenthood is not necessarily associated with lower marital satisfaction in couples. One element that appears to support the maintenance of a positive marital relationship is the relationship between the mother and the father in their role as parents, that is, their coparenting relationship or alliance. Coparenting alliance is defined as ways in which parents support or undermine each other in their role as parents (16). Le et al. (15) reported that the levels of women's perceived coparenting alliance when their child was 6 months old predicted their evaluation of the quality of their relationship with their partner when the child was 3 years old. Likewise, Don and colleagues (14) reported that perceptions of the coparenting alliance, as reported by mothers of children aged 4 months, were a significant predictor of maternal relationship satisfaction when their children were 9 months of age. The coparenting alliance has also been associated with maternal well-being. For example, Schoppe-Sullivan and colleagues (13) determined that mothers who perceived greater supportive coparenting when their child was 3 months old experienced lower levels of parenting stress when the child was 9 months old.

In the present study, in addition to exploring the hypothesis mentioned above, we further sought to determine whether the same patterns of mediational relationships could be found in both mothers whose youngest child was aged 24 months or less and those whose youngest child was aged 25 months or older. Based on past studies, we believe it is important to distinguish between the first 2 years of parenting and later childhood. Indeed, past studies suggest that even though chronicity of maternal depression is highly important, depression emerging in the first few years after birth can greatly influence children's outcomes (6, 23). Thus, when studying the antecedents of mothers' mental health and well-being, it appears important to distinguish between the initial postpartum period (i.e., first 2 years) and the later childhood period.

## Materials and Methods

### Participants and Procedure

A descriptive correlational study was conducted with a sample of 447 mothers recruited from 2013 to 2016 across Quebec, Canada, through key informants and social media messages. Key informants in community and healthcare organizations informed mothers about the research project through pamphlets. To participate, mothers needed to be the biological mother of a child <5 years old and be able to read and understand French. Mothers in the sample had a mean age of 29.43 years (SD = 4.48, min = 18, max = 44), and the mean age of their youngest child's father was 32.36 years (SD = 5.89, min = 19, max = 66). Most mothers were born in Canada (93.7%), had at least a post-secondary level of education (79.6%), and had a household income of $70,000 or more (58.6%, *n* = 262). In terms of employment, 41.2% of the mothers had a full-time job or were self-employed, 12.1% had a part-time job, 16.8% were either students or unemployed, and 26.0% were on maternity leave. Forty per cent of the mothers were married, and 60% were in common-law unions. The age of their youngest child, at the time they completed the questionnaire, varied from <1 to 72 months (*M* = 22.2 months, *Med* = 18.0, *SD* = 17.0). The mothers had from one to five children each (*M* = 1.9, *Med* = 2, *SD* = 0.9). This sample was comparable in profile to previous studies we have carried out in Quebec (Canada) with francophone parents (24, 25). In Quebec, from 2013 to 2016, roughly 37% of children were born to parents who were married, with most born to two parents in a common-law union (26). For the same time period, 64, 6% to 67, 3% of women aged 25–34 years in Quebec held a post-secondary degree (college or university) (27) and the average income of two-parent households with children ranged from 112,700 to 118,600$ (28). Thus, the present sample is slightly more educated than women of the same age in Quebec. There were no significant demographic differences between the two groups of mothers (0–24 months and 25+ months). Of specific interest is the confirmation that mothers in each group had, on average, the same number of children (*M* = 1.84 and 1.89 respectively, Mann-Whitney *U* standardized test = 1.61, *p* = 0.11). The mean age of mothers' youngest child was 11.2 months (SD = 7.0) in the 0–24 months group and 40.3 months (SD = 12.6) in the 25+ months group [*t*_(230.72)_ = 27.51, *p* < 0001, adjusted for unequal variances]. To reach a less advantaged population, participants were given the option of filling in the questionnaire online or on paper, as they preferred. Questionnaires took about 45 min to complete.

Protection of human participants was reviewed and approved by the institutional review boards of the Université du Québec (CER-12-184-04-04.01) and the participating medical centers. All participants provided informed consent prior to participating in the research. Online participants needed to give online consent prior to accessing the questionnaires. All participants had the right to withdraw from the study at any time without prejudice. The dataset used and analyzed during the current study is available from the corresponding author on reasonable request.

### Measures

*Perceived paternal involvement*. Participants completed a measure of paternal involvement (29), which is composed of 52 items reflecting tasks performed by fathers with respect to their children. Sample items are that the father “puts your child to bed at night” and “comforts your child when he or she cries.” Answers are provided on a scale of 1 (never) to 6 (every day). Total score of the scale is obtained by computing the average of the items. A higher score indicates a greater degree of paternal involvement as perceived by the mother. The internal consistency of this scale for the present sample was excellent, with a Cronbach's alpha of 0.94.

Mothers' satisfaction with their relationship with their partner was assessed for two distinct relational dimensions: coparental and marital relationships. *Coparenting alliance*. Participants completed the Parenting Alliance Inventory (30). This instrument consists of 20 items on a 5-point Likert scale ranging from 1 (strongly disagree) to 5 (strongly agree) measuring the extent to which the partners form a team to perform the various tasks associated with parenting. A sample statement is, “My spouse tells me I am a good parent.” The total score of the scale is obtained by computing the average of the 20 items. Thus, scores can vary between 1 and 5, with higher scores indicating stronger coparenting alliance. The internal consistency of this scale for the present sample was excellent, with a Cronbach's alpha of 0.96.

*Dyadic adjustment*. To assess the quality of the marital relationship, participants completed a short version of the Dyadic Adjustment Scale (31) translated into French by Sabourin et al. (32), composed of four statements with responses ranging from 0 (never) to 5 (always). A sample statement is, “Do you confide in your partner?” The total score of the scale is obtained by adding up the scores (range 0 to 20) on the statements: the higher the score, the better the quality of the marital relationship. The internal consistency of this scale for the present sample was good, with a Cronbach's alpha of 0.85.

Mothers' mental health and well-being was examined with measures of depression and parenting stress. *Depression*. Mothers' current level of depressed mood was assessed with the Beck Depression Inventory [BDI, Beck et al. (33)]. This measure is composed of 21 items answered on a 4-point scale representing increasing levels of depressed behaviors (0 = I don't cry more than before, to 3 = I would like to cry but I'm not able to). The internal consistency of this scale for the present sample was good, with a Cronbach's alpha of 0.88.

*Parenting stress*. Participants completed the 36-item Parenting Stress Index [PSI (34) translated into French by Bigras et al. (35)]. This measure reflects the level of stress felt by parents regarding their parenting role, their child's temperament, and their interactions with their child. The measure is divided into three subscales, each containing 12 items: parenting distress (present sample Cronbach's alpha = 0.83), difficult child (present sample Cronbach's alpha = 0.87), and parent–child dysfunctional interactions (present sample Cronbach's alpha = 0.82). Sample statements for the different subscales include: parenting distress— “I often have the feeling that I cannot handle things very well;” difficult child— “My child doesn't seem to smile as much as most children;” parent-child dysfunctional interactions– “My child's behavior is more of a problem than I expected.” Scores on each subscale can range from 12 to 60, with a higher score indicating more stress.

### Data Analysis

Descriptive analyses were performed to ensure that all variables were normally distributed. Missing data, which constituted <10% of the data, were replaced using the mean of each variable. Multivariate and univariate analyses of variance were also conducted to detect differences on each variable between the two groups of mothers (divided according to age of youngest child, see below). IBM Statistical Package for the Social Sciences (SPSS) for Windows, version 20.0 (IBM Corp. 2011) was used for these analyses. Path modeling analyses with observed scores were performed using LISREL 8.80 (36). The analyses were conducted with the covariance matrix using the maximum likelihood estimation procedure. The goodness-of-fit indices selected to determine the adequate fit of the data to the models were the chi-square goodness-of-fit statistic; the Root Mean Square Error of Approximation (RMSEA) and its 90% confidence interval; the Normed Fit Index (NFI); the Non-Normed Fit Index (NNFI); the Comparative Fit Index (CFI); the Goodness of Fit Index (GFI), and the Standardized Root Mean Square Residuals (SRMR) (37). The following goodness-of-fit guidelines were followed: a non-significant chi-square statistic, NFI, NNFI, CFI and GFI indices above 0.90 (38), a RMSEA below 0.08 and the upper limit of its 90% confidence interval smaller than 0.10, (39) and, finally, a SRMR below 0.05.

Multi-sample analyses were then conducted to determine whether the proposed path model was valid across subgroups based on the age of participants' youngest child. To properly conduct multi-group analyses, the following four-step procedure was followed: (1) test the selected model with the total sample; (2) test the multi-group model specifying that the same patterns of associations must be found in all subgroups, yet with all parameters estimated freely, thus allowing estimates to vary between groups; (3) test the multi-group model specifying that the associations are invariant in all subgroups (i.e., parameter estimates constrained to be equal across groups); and (4) compute the chi-square difference between the multi-group path model's chi-squares and the chi-square of the final model with the entire sample. A non-significant chi-square difference demonstrates that the same patterns can be found in all subgroups or, in the case of the test of invariance model, that the relationships are invariant between the two groups [see Deng et al. (40)]. For all models, indirect effects and 95% confidence intervals were estimated using bias corrected bootstrapped confidence intervals based on 5,000 samples, using the PROCESS macro for SPSS version 3.2.

## Results

### Observed Differences Based on the Age of Participants' Youngest Child

Two groups were created based on the age of participants' youngest child. Building on previous research, we created a first group composed of the mothers of children between 0 and 24 months of age and a second group of mothers of children aged 25 months and older. A multivariate analysis of variance (MANOVA) was conducted on key study variables, followed by univariate analyses of variance (ANOVAs), to determine whether the two groups differed on the study's variables. The MANOVA indicated that the mothers whose youngest child was 0–24 months of age fared slightly better overall on study variables than those whose youngest child was 25 months or older [*F*_(7,439)_ = 4.36, *p* = 0.0001, partial η^2^ = 0.065]. Results for univariate ANOVAs are presented in Table 1. These more specifically suggested that perceived paternal involvement, parenting alliance, and dyadic adjustment were significantly higher in mothers of younger children and that two subscales of parenting stress (parent-child dysfunctional interaction and difficult child) were significantly higher in the mothers of the older children, although effect sizes were generally small (η^2^ of 0.01 is a small effect whereas and η^2^ of 0.06 is a medium sized effect according to Cohen (41).

**Table 1:** Descriptive statistics and univariate analyses of ANOVAs comparing two groups of mothers distinguished on the basis of age of youngest child.

	**Youngest child aged**				
	**0–24 months** ***n* = 279**	**25+ months** ***n* = 168**	**Univariate ANOVA**		
	***M* (SD)**	***M* (SD)**	***F*_**(1, 445)**_**	***p***	**Partial η^**2**^**
PSI parenting distress	28.19 (8.08)	28.46 (8.37)	0.11	0.74	0.00
PSI parent-child dysfunctional interactions	18.52 (5.68)	19.79 (5.51)	5.36	0.02	0.01
PSI difficult child	22.09 (7.88)	24.85 (8.70)	11.91	0.001	0.03
BDI depression	8.62 (7.08)	8.52 (7.18)	0.02	0.89	0.00
Dyadic adjustment	16.56 (2.69)	15.61 (3.09)	11.34	0.001	0.03
Coparental alliance	0.70 (0.18)	0.66 (0.18)	5.03	0.03	0.01
Perceived paternal involvement	4.07 (0.61)	3.94 (0.55)	5.04	0.02	0.01

*PSI, Parenting Stress Index; BDI, Beck Depression Inventory*.

### Path Model for Total Sample and for Groups of Mothers Distinguished by Age of Youngest Child

The path model tested in the present study was composed of nine observed variables; three exogenous observed variables (perceived paternal involvement, household income, youngest child's age) and six endogenous variables (dyadic adjustment, coparenting alliance, depression, and the three subscales of parenting stress). Table 2 presents the correlations between study variables for each of the two groups distinguished by age of mothers' youngest child (0–24 or 25+ months). In both groups, correlations between key model variables are in the expected direction, of medium to large effect size (41) and nearly all attain significance (*p* < 0.05). Age of the youngest child and household income are generally not significantly associated with the key study variables within each group. This suggest that the residual variability in the youngest child's age with groups is unrelated to the key variables, whereas household income only modestly correlates with two of the seven key variables.

**Table 2:** Correlations between variables for two groups of mothers distinguished on the basis of age of youngest child.

									**Youngest child 25+ months**
**Variables**	**1**	**2**	**3**	**4**	**5**	**6**	**7**	**8**	**9**
PSI parenting distress	-	0.43^***^	0.41^***^	0.64^***^	–0.41^***^	–0.46^***^	–0.44^***^	–0.04	–0.09
PSI parent-child dysfunctional interaction	0.45^***^	-	0.67^***^	0.34^***^	–0.13	−0.24^***^	−0.17^*^	0.05	−0.21^**^
PSI difficult child	0.45^***^	0.66^***^	-	0.36^***^	−0.24^**^	−0.39^***^	−0.26^***^	−0.11	−0.18^*^
BDI depression	0.58^***^	0.33^***^	0.35^***^	-	−0.35^***^	−0.36^***^	−0.36^***^	0.03	−0.14
Dyadic adjustment	−0.44^***^	−0.14^*^	−0.13^*^	−0.34^***^	-	0.61^***^	0.35^***^	0.08	0.08
Coparental alliance	−0.50^***^	−0.26^***^	−0.24^***^	−0.26^***^	0.62^***^	-	0.51^***^	0.10	0.05
Perceived paternal involvement	−0.40^***^	−0.18^***^	−0.15^**^	−0.21^***^	0.51^***^	0.51^***^	-	−0.05	0.06
Age of youngest child in months	−0.03	−0.07	0.05	−0.10	0.00	0.10	−0.00	-	0.09
Household income	−0.11	−0.10	0.02	−0.03	0.07	0.05	0.10	−0.06	-
Youngest child 0–24 months									

Household income: 1 = < $70,000, 2 = equal or more than $70,000. *p < 0.05, **p < 0.01, ***p < 0.001.

The first model tested the hypothesis that dyadic adjustment and coparenting alliance mediate the associations between perceived paternal involvement and the four maternal outcome variables, that is maternal depression and each of the three parental stress subscales. Covariances were specified between the two mediators as well as between the four outcome variables. The model had a satisfactory fit to the data [χ2 (*df* = 16, *n* = 451) = 39.93, *p* = 0*.0*01, RMSEA = 0.06 (0.03; 0.08), NFI = 0.98, NNFI = 0.97, CFI = 0.98, GFI = 0.98 and SRMR = 0.04]. Inspection of the standardized results indicated non-significant paths between dyadic adjustment and two parenting stress subscales: parent-child dysfunctional interactions and difficult child. A second model was tested in which these two parameters were fixed at zero (paths removed). This second model also adequately fit the data [χ2 (*df* = 18, *n* = 451) = 39.44, *p* = 0.003, RMSEA = 0.05 (0.03; 0.07), NFI = 0.98, NNFI = 0.97, CFI = 0.99, GFI = 0.98 and SRMR = 0.04]. Although not very different from the initial model, this second model was preferred because all estimated paths were found to be significant and because the inspection of the residual matrix did not suggest any additional significant relationships. This solution was retained as the total sample model (Table 3).

**Table 3:** Goodness-of-fit indices of all models.

	**Model chi-square**				**RMSEA (90% CI)**	**NFI**	**NNFI**	**CFI**	**GFI**	**SRMR**
**Model**	**χ^**2**^**	***df***	***n***	***p***						
Total sample	39.44	18	451	0.003	0.05 (0.03; 0.07)	0.98	0.97	0.99	0.98	0.04
Group A: 0–24 months	23.02	18	280	0.19	0.03 (0.00; 0.07)	0.98	0.99	0.99	0.98	0.04
Group B: 25+ months	33.16	18	171	0.02	0.07 (0.03; 0.11)	0.95	0.94	0.97	0.96	0.07
Multi-sample: Same pattern (parameters varying between models)	57.95	36	451	0.02	0.05 (0.03; 0.08)	0.96	0.97	0.98		
Multi-sample: Invariance (parameters constrained to be equal across groups)	175.25	63	451	0.0001	0.09 (0.07; 0.11)	0.90	0.92	0.93		
**Model comparisons**	**Δχ^2^**	**Δ*****df***		***p***						
M_totalsample_ – M_samepattern_	18.51	18		0.42						
M_totalsample_ – M_invariance_	135.81	45		<0.0001						
M_samepattern_ – M_invariance_	117.30	27		<0.0001						

**Figure 1:**
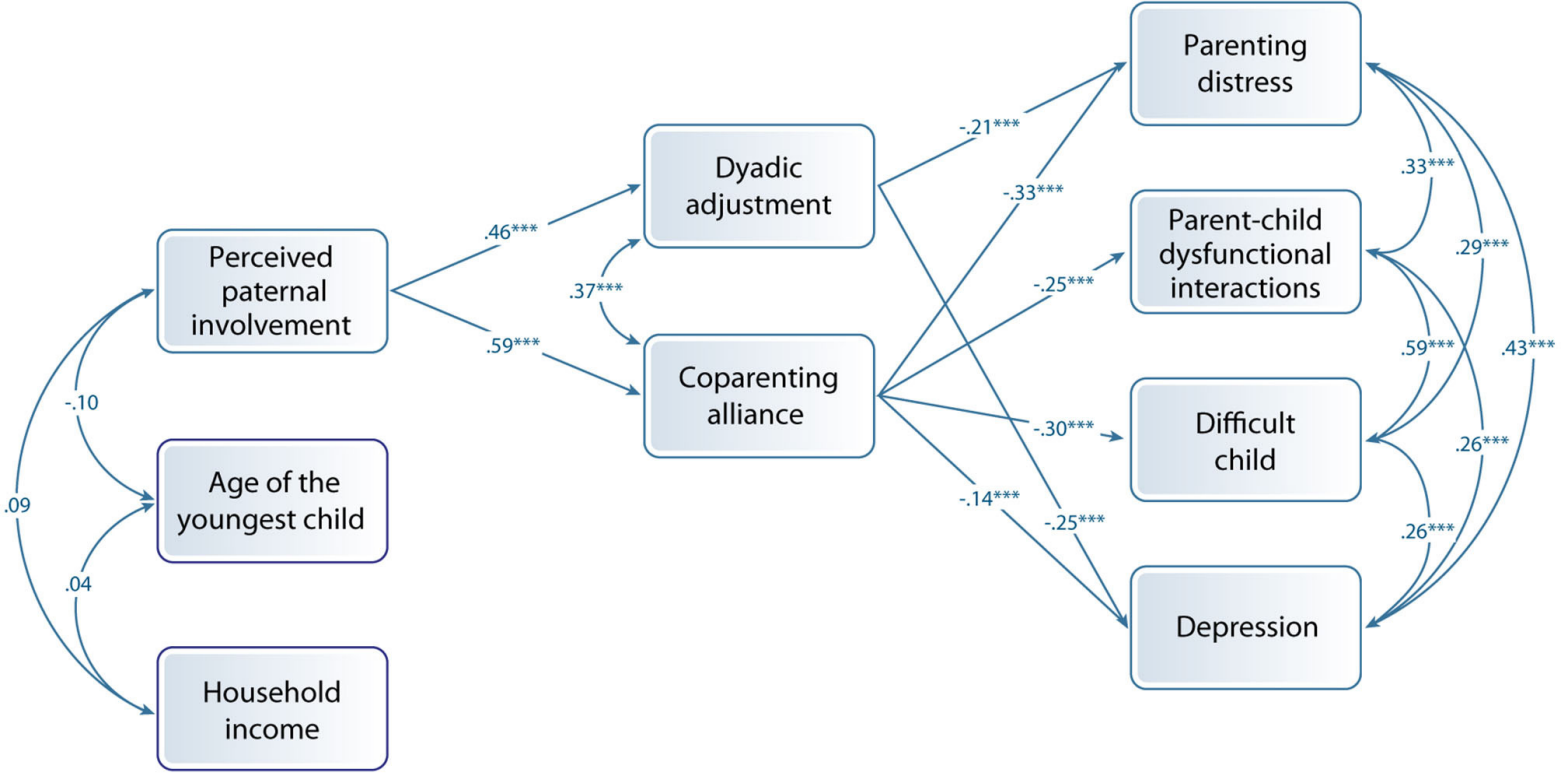
Model with the complete sample of mothers (total sample).

*Same pattern = Path model illustrated in Figure 1 fitted to both groups simultaneously, allowing estimates to vary between groups (i.e., within-group parameters estimated). Invariance = Path model illustrated in Figure 1 fitted to both groups simultaneously, with model estimates constrained to be equal across groups (i.e., common parameters estimated for both groups)*.

Results of the total sample are presented in Figure 1. Perceived paternal involvement positively and significantly explained both dyadic adjustment and coparenting alliance, but direct paths to maternal depression and parental stress subscales were not suggested. In turn, dyadic adjustment negatively and significantly accounted for parenting distress and mothers' depression, while coparenting alliance negatively and significantly accounted for all three subscales of parenting stress and mothers' depression.

To determine whether associations between model variables differed between mothers whose youngest child was aged between 0 and 24 months (group A) and mothers whose youngest child was 25 months or older (group B), multi-group models were tested. Table 3 shows the results of multi-group analyses. The total sample model presented good fit to the data for each group tested separately. Furthermore, the chi-square difference statistic between the model fitted to the entire sample (total sample) and the multi-group same patterns model (varying parameter between models) was found to be non-significant. However, the chi-square difference statistic between the models fitted the entire sample, and the multi-group invariant model (with parameters constrained to be equal across groups) was found to be significant. Results indicated that the same patterns of associations between study variables was present in the two groups but that the strength of these associations should be considered different between groups. Thus, the same model was fitted to groups A and B separately, allowing parameter estimates to vary freely for each group. Parameter estimates for the path model fitted to the total sample, as well as separately to each group of mothers, are presented in Table 4.

**Table 4:** Parameter estimates for the path model (illustrated in Figure 1) fitted to the total sample and two distinct subsamples distinguished based on age of mothers' youngest child (multi-sample: same pattern).

**Parameter estimated**	**Total sample**		**Group A 0–24 months**		**Group B 25+ months**	
*Correlations*	σ	*p*	σ	*p*	σ	*p*
Pat Inv ↔ Young Child	***–*****0.10**	0.03	−0.00	0.47	−0.06	0.07
Pat Inv ↔ Income	0.09	0.27	0.11	0.06	0.06	0.23
Young Child ↔ Income	0.04	0.10	−0.05	0.16	0.12	0.12
Dyad Adj ↔ Copar Alli	**0.37**	<0.0001	**0.27**	<0.0001	**0.46**	<0.0001
Par Dist ↔ Dysf Int	**0.33**	<0.0001	**0.32**	<0.0001	**0.33**	<0.0001
Par Dist ↔ Diff Child	**0.29**	<0.0001	**0.32**	<0.0001	**0.26**	<0.001
Par Dist ↔ Depress	**0.43**	<0.0001	**0.40**	<0.0001	**0.46**	<0.0001
Dysf Int ↔ Diff Child	**0.59**	<0.0001	**0.58**	<0.0001	**0.61**	<0.0001
Dysf Int ↔ Depress	**0.26**	<0.0001	**0.27**	<0.0001	**0.26**	<0.001
Diff Child ↔ Depress	**0.26**	<0.0001	**0.28**	<0.0001	**0.23**	<0.01
*Regression weights*	β	*p*	β	*p*	β	*p*
Pat Inv → Dyad Adj	**0.46**	<0.0001	**0.47**	<0.0001	**0.40**	<0.0001
Pat Inv → Copar Alli	**0.59**	<0.0001	**0.62**	<0.0001	**0.54**	<0.0001
Dyad Adj → Par Dist	**−0.21**	<0.001	**−0.22**	<0.0001	**−0.20**	<0.01
Dyad Adj → Depress	**−0.25**	<0.0001	**−0.33**	<0.0001	**−0.20**	<0.01
Copar Alli → Par Dist	**−0.33**	<0.0001	**−0.35**	<0.0001	**−0.33**	<0.001
Copar Alli → Dysf Int	**−0.25**	<0.0001	**−0.26**	<0.0001	**−0.23**	<0.001
Copar Alli → Diff Child	**−0.30**	<0.0001	**−0.23**	<0.0001	**−0.41**	<0.0001
Copar Alli → Depress	**−0.14**	0.01	**−0.07**	0.18	**−0.23**	<0.01
*Variance explained (R^2^)*						
Dyad Adj	0.21		0.26		0.12	
Copar Alli	0.35		0.41		0.26	
Par Dist	0.09		0.12		0.06	
Dysf Int	0.02		0.03		0.01	
Diff Child	0.03		0.02		0.04	
Depress	0.04		0.04		0.04	
*Indirect effects of Pat Inv*		95% CI		95% CI		95% CI
→ Dyad Adj → Par Dist	**−0.14**	(−0.18; −0.09)	**−0.17**	(−0.23; −0.10)	**−0.11**	(−0.17; −0.05)
→ Dyad Adj → Depress	**−0.13**	(−0.18; −0.08)	**−0.17**	(−0.25; −0.08)	**−0.09**	(−0.16; −0.03)
→ Copar Alli → Par Dist	**−0.22**	(−0.28; −0.16)	**−0.29**	(−0.38; −0.20)	**−0.19**	(−0.29; −0.10)
→ Copar Alli → Dysf Int	**−0.14**	(−0.21; −0.08)	**−0.20**	(−0.29; −0.11)	**−0.14**	(−0.26; −0.03)
→ Copar Alli → Diff Child	**−0.18**	(−0.25; −0.11)	**−0.19**	(−0.29; −0.09)	**−0.22**	(−0.34; −0.11)
→ Copar Alli → Depress	**−0.13**	(−0.20; −0.07)	**−0.14**	(−0.25; −0.05)	**−0.17**	(−0.27; −0.07)

*Group A = Mothers whose youngest child is aged 0–24 months. Group B = Mothers whose youngest child is aged 25+ months. Pat Inv = Perceived Paternal involvement. Young Child = Age of youngest child. Income = Household income. Par Dist = Parental distress. Dysf Int = Parent-child dysfunctional interactions. Diff Child = Difficult child. Depress = Depression. Dyad Adj = Dyadic adjustment. Copar Alli = Coparenting Alliance. 95% CI = bias corrected bootstrapped 95% confidence interval. Parameter estimates in bold can be deemed different from zero (p < 0.05)*.

Comparison of parameter estimates for regression weights for each group revealed a few more salient differences between the two groups. Regression parameters suggested that perceived paternal involvement more strongly accounted for the dyadic adjustment and coparenting alliance of mothers of younger children, that dyadic adjustment more strongly accounted for depression in these mothers, but, inversely, that coparenting alliance more weakly accounted for these mothers' appraisal of child difficultness and reports of depression. Indeed, the latter association could be considered null for mothers whose youngest child was aged 0–24 months, but significant and negative for mothers of older children.

### Indirect Contribution of Perceived Paternal Involvement to Explaining Maternal Depression and Parental Stress

The indirect effects and the bias corrected bootstrapped 95% confidence intervals for indirect contributions of perceived paternal involvement account for mothers' parental stress, as assessed sub-scales of the Parental Stress Index (parental distress, dysfunctional interactions and child difficultness), and depressive symptoms on the Beck Depression Inventory. In the total sample model, as well as in those estimated for each of the two groups of mothers, lower perceived paternal involvement was found to significantly account for poorer maternal outcomes on all four indicators indirectly through its associations with both dyadic adjustment and coparenting alliance (i.e., their 95% confidence interval did not include zero).

## Discussion

Given the significant impact that being a parent has on mothers' psychological mental health and well-being (42–44), and given the large body of research indicating the significant short- and long-term impacts that maternal stress, anxiety and depression can have on children (1–6), we sought to identify factors which may account for maternal mental health and stress. Some research has investigated the contribution of partner support (7, 9–11) as well as of general support (8) to easing maternal parenting stress. Other research has focused on the benefits of paternal involvement with childcare responsibilities for maternal parenting stress (12, 16). Further work has reported associations between paternal involvement and marital satisfaction (14–16, 22), as well as between marital satisfaction and maternal mental health (19–21). However, to the best of our knowledge, no previous work has attempted to bring all these elements within the same explanatory model.

We therefore hypothesized that mothers' perception of greater paternal involvement with childcare responsibilities contributes to explain better maternal mental health and well-being, through its association with two dimensions of mothers' relationship with their partner: dyadic adjustment and coparenting alliance. Overall, the results of the present study support our hypothesis. Mothers' perceptions of paternal involvement were found to be strongly and significantly related to both dyadic adjustment and coparenting alliance. In turn, dyadic adjustment significantly accounted for parenting distress and depression, while coparenting alliance significantly contributed to explaining all parenting stress subscales as well as maternal depression. Indirect associations from perceptions of paternal involvement to all four maternal outcomes were moderate in size but all different from zero.

It is thus important to consider mothers' perceptions of paternal involvement with childcare responsibilities as a dimension of parenting that can account for maternal mental health and well-being, as shown in the present study and previous work (12, 16), as well as a notable contributor to children's emotional and behavioral outcomes (45–48). A recent study has demonstrated, with longitudinal data from pregnancy to child's age of 24 months, that changes in fathers' perception of the quality of their marriage is an important factor in accounting for coparenting quality (49). That study reported, for instance, that a decline in fathers' marital satisfaction over the first 2 years of their infant's life predicted lower involvement in parenting, while an increase in marital conflict predicted lower cooperative coparenting. Thus, fathers' perception of the quality of their relationship over the first few years, and probably later as well, appears key to understanding their involvement in childcare responsibilities. This is in line with past theory and research, which suggest a bidirectional relationship between coparenting and marital functioning (50–52). Le et al. (15) showed that men's and women's perceptions of their relationship quality during pregnancy positively predicted their perceptions of coparenting support at 6 months postpartum. Similarly, men's and women's perceptions of their relationship quality at 6 months postpartum positively predicted their perceptions of coparenting support 3 years postpartum. However, only for women was perceived coparenting support at 6 months postpartum significantly related to relationship quality at 3 years postpartum, which is in line with the present results. It thus appears that the patterns of relationships between coparenting support and marital satisfaction are slightly different for men and women. Future research should investigate this issue further and replicate the present study results with data from both mothers and fathers.

Based on previous work which suggested that the initial postpartum period and the later childhood period differ in terms of the impacts of maternal mental health and well-being on children's emotional and behavioral problems (6, 23), we further sought to test the proposed mediational model separately between mothers whose youngest child was aged 0–24 months (Group A) and mothers whose youngest child was aged 25 months and older (Group B). First, the present results from analyses of variance indicated that the mothers in Group A perceived greater paternal involvement, higher dyadic adjustment, and higher coparental alliance than did those in Group B. Potentially related to these differences, the mothers in Group A also reported less parenting stress. Results of the multi-sample path models indicated that the two groups presented the same global pattern of associations, with slight differences between the groups. For instance, the association between perceived paternal involvement and each of the two dimensions of relationship satisfaction appeared stronger in the mothers of Group A. Furthermore, coparenting alliance contributed significantly to explaining depressive symptoms in mothers in Group B, but not those in Group A. Thus, when investigating predictors of maternal mental health and well-being, it appears warranted to distinguish between the experiences of mothers of infants and young toddlers and those of mothers of older children.

### Strengths and Limitations

Although path modeling enables testing of mediational models for multiple correlated outcome variables, even considering parallel mediations, it is not a substitute for an experimental research design or even more internally valid longitudinal research. Because of the cross-sectional correlational design of the present study, no causal conclusions can be drawn from its results. Future prospective longitudinal studies are needed to replicate the proposed mediational model. Additionally, experimental research on the effects of interventions aimed at stimulating father involvement, with control group comparisons, and in which data are collected from both fathers and mothers, would serve to support the hypothesized causal role of father involvement with regard to parental well-being. However, the sample of the present study was very large, included mothers whose youngest child was aged between newborn and 5 years old (median 22 months), was representative of the type of union between parents of young children in Québec (predominantly common-law), and included families of diverse income levels (although the mean household income was lower than the provincial mean for two-parent families with children). Thus, the present results reflect the experience of a large range of mothers, albeit with a sample of mother that are, as is the case in much research with volunteer participants, slightly more educated than the norm for women in Quebec. The broad distinction of mothers into two groups based on the age of their youngest child does not allow the finer analysis of mental health problems, such as perinatal depression which is diagnosed up to 4 weeks following childbirth (53). Such time specific considerations would be more appropriately examined through longitudinal research.

Another limitation to the present study is the self-reported nature of all measures. Although all questionnaires used are theoretically valid and their psychometric properties have been demonstrated previously, it is possible that mothers answered in a socially desirable manner. It would be important for future research to use other sources of data, such as observations of fathers' involvement and coparenting dynamics. This might be all the more relevant given that research has suggested that depressed women have a tendency to feel less supported than they really are (54).

Finally, the present study was conducted with data collected solely from mothers and reflecting their perceptions of fathers' involvement. Future work including data from fathers and possibly adopting a more objective or at least more balanced report of father involvement by considering fathers' perceptions, would be highly important to better understand marital and coparenting dynamics and how they may account for both maternal and paternal mental health and well-being.

### Clinical Implications

The present study, aimed at better understanding the factors accounting for maternal depression and parental stress, has identified elements that can be: (1) used to identify vulnerable mothers at an early stage and (2) acted upon in an intervention effort. Past research has indicated that maternal anxiety and depression influence the quality of the mother–child relationship (55, 56). Thus, interventions that are supportive of both parents, encourage paternal involvement with childcare responsibilities, and are attentive to the quality of the relationship between parents, whether relative to conjugal or to coparenting dimensions, could be of great help to children through potentially greater mental health and well-being of parents, specifically mothers. A recent meta-analysis of the efficacy of different psychological therapies for postnatal depression found that all types of interventions, when compared with controls, were similarly effective in reducing depression in new mothers (57). It further reported positive impacts on adjustment to parenthood, marital relationship, social support, stress, and anxiety. Thus, in line with the present study, identifying mothers in difficulty—those who are vulnerable, who perceive less involvement from their partner, who are in difficult or unsatisfactory marital relationship, or who are experiencing high levels of stress, depression, and anxiety—in primary care settings and offering effective psychological support or therapies could ultimately be beneficial for mothers, fathers and children alike. Furthermore, the results of the present study underscore the likely benefits for families of father inclusive preventative programs and interventions, specifically tailored to helping fathers be more involved such as the Father Friendly Initiative (58, 59). Group interventions such as Present Fathers, Successful Children have been found to foster better father–child relationships (60, 61) and could be beneficial for mothers' mental health.

## Conclusion

The results of the present study help shed light on familial and relational factors associated with better mental health and lower parental stress in mothers of young children. Specifically, it was found that the more mothers perceive their partner as involved with childcare responsibilities, the less they experience depressive symptoms and stress regarding their parenting responsibilities. Further, the present results constitute a first demonstration of the mediational role of mothers' satisfaction with their conjugal and coparenting relationship with their partner in the association between parental involvement and maternal depression and parental stress. Future research is necessary to confirm the present results and to test the hypothesized causal role of paternal involvement with a more internally valid research design, but the paternal and relational factors associated with maternal mental health and well-being identified in both this and prior research appear highly useful to inform father inclusive psychological interventions designed for mothers and fathers.

## Data Availability Statement

The datasets analyzed for this study can be found in the private repository of the Father Friendly Initiative at Université du Québec en Outaouais. The datasets can be made available upon request to the first author. Data from: Father Friendly Initiative. Université du Québec. (2018).

## Ethics Statement

Protection of human participants was approved by the institutional review.

boards of the Université du Québec (CER-12-184-04-04.01) and the participating medical.

centers. All participants provided informed consent prior to participating in the research. Online

participants needed to give online consent prior to accessing the questionnaires.

## Author Contributions

FdM and CG were responsible for planning the study, securing funding and carrying out the data collection. TP was responsible for planning the data analysis. GL performed the data analysis and wrote a first draft of the manuscript. All authors contributed to the revision of the manuscript and the production of the final version. All authors contributed to the article and approved the submitted version.

## Conflict of Interest

The authors declare that the research was conducted in the absence of any commercial or financial relationships that could be construed as a potential conflict of interest.
